# Assessing the impact of a test and vaccinate or remove badger intervention project on bovine tuberculosis levels in cattle herds

**DOI:** 10.1017/S0950268823001061

**Published:** 2023-07-04

**Authors:** Liam Patrick Doyle, Alan W. Gordon, Colm Molloy, Maria J. H. O’Hagan, Anastasia Georgaki, Emily A. Courcier, Roly G. Harwood, Fraser Duncan Menzies

**Affiliations:** 1 Veterinary Epidemiology Unit, Department of Agriculture, Environment and Rural Affairs, Belfast, UK; 2 Statistical Services Branch, Agri-Food and Biosciences Institute, Belfast, UK; 3 Land and Property Services, Department of Finance, Belfast, UK

**Keywords:** bovine tuberculosis, cattle, epidemiology, Eurasian badgers (*Meles meles*), *Mycobacterium bovis*, remove (TVR), test, vaccinate

## Abstract

Bovine tuberculosis (bTB) is a chronic, zoonotic infection of domestic and wild animals caused mainly by *Mycobacterium bovis.* The Test and Vaccinate or Remove (TVR) project was a 5-year intervention (2014–2018) applied to Eurasian badgers (*Meles meles*) in a 100 km^2^ area of County Down, Northern Ireland. This observational study used routine bTB surveillance data of cattle to determine if the TVR intervention had any effect in reducing the infection at a herd level. The study design included the TVR treatment area (Banbridge) compared to the three adjacent 100 km^2^ areas (Dromore, Ballynahinch, and Castlewellan) which did not receive any badger intervention. Results showed that there were statistically lower bTB herd incidence rate ratios in the Banbridge TVR area compared to two of the other three comparison areas, but with bTB herd history and number of bTB infected cattle being the main explanatory variables along with Year. This finding is consistent with other study results conducted as part of the TVR project that suggested that the main transmission route for bTB in the area was cattle-to-cattle spread. This potentially makes any wildlife intervention in the TVR area of less relevance to bTB levels in cattle. It must also be noted that the scientific power of the TVR study (76%) was below the recommended 80%, meaning that results must be interpreted with caution. Even though statistical significance was achieved in two cattle-related risk factors, other potential risk factors may have also demonstrated significance in a larger study.

## Introduction

*M. bovis* infection in cattle (bovine tuberculosis (bTB)) and Eurasian badgers (*M. meles*) is a persistent and costly problem for the farming industries and governments of the United Kingdom (UK) and Republic of Ireland (ROI) [1]. Evidence of an association between *M. bovis* infection in cattle and badgers exists from a variety of sources. In ROI, the role of the badger in maintaining persistent infection in cattle herds was examined in the east Offaly (1989–1995) and in four area (1997–2002) field trials, where proactively culled areas were compared to matched reference areas in which reactive culling was carried out [2–5]. In Great Britain (GB), a field trial called the Randomized Badger Culling Trial (RBCT) was carried out between 1998 and 2006, which compared areas of proactive culling to matched areas in which there was no badger culling [6]. The overall results of both the ROI and the GB work showed that levels of bTB in cattle were lower in areas subjected to extensive proactive badger culling compared to matched control areas [4]. More recently in GB (2013–2017), private industry–led proactive badger culling was assessed for its effects on bTB herd incidence, where licensed culling areas were matched to comparison areas. The results of this work over a 4-year period showed that there was a reduction in bTB herd incidence in two of the three study areas [7, 8]. Other important evidence which supports an association between *M. bovis* infection of cattle and badgers in a locality is molecular genotyping, where GB and Irish studies show that cattle and badgers tend to share the same genotypes in the same areas [9–11]. In relation to bTB, cattle and badgers exist in what could be described as a multi-host system with bi-directional transmission of infection [12–16]. Current evidence on interactions between badgers and cattle indicates that direct contact between the species capable of providing an opportunity for aerosol spread of infection is a very rare event [17–20]. Rather, fomite transfer, in which badgers and cattle use the same space but at different times (indirect spread), may result in the potential transfer of infectious material [19–22]. It must also be noted that the two-host bTB transmission system between badgers and cattle is a complex interaction probably varying between different localities depending on a range of badger and cattle metrics unique to a particular area, but with higher transmission rates within species than between species [12–15].

The Test and Vaccinate or Remove (TVR) project was a 5-year badger intervention (2014–2018) in a 100 km^2^ area of County Down, Northern Ireland [23]. During the TVR project, badgers were captured annually in cages, pen-side tested for bTB, and, based on the real-time test results, either euthanised (test-positive) or vaccinated pre-release (test-negative). Captured badgers also had a range of physical characteristics recorded and clinical samples collected, and, in parallel work, some had Global Positioning System (GPS) collars fitted to collect location/time data [24]. A Bayesian analysis of the test results from badgers caught in the TVR area over the 5-year period demonstrated a significant reduction in *M. bovis* prevalence in badgers from 14% to 1.9% [25].

## Study objective

The primary objective of this study was to assess if the application of a TVR intervention in the 100 km^2^ core area had an impact on the level of bTB in the local cattle herds compared with similar-sized non-treatment areas in the same locality over the same time period. It was also an objective of this study to determine if the application of TVR intervention led to any increase in bTB among herds adjacent to the treatment area (perturbation effect). This part of the objective was met by comparing the bTB herd incidence in a 2 km buffer zone around the TVR intervention with similar buffer zones around the comparison areas.

## Materials and methods

**Figure 1.**
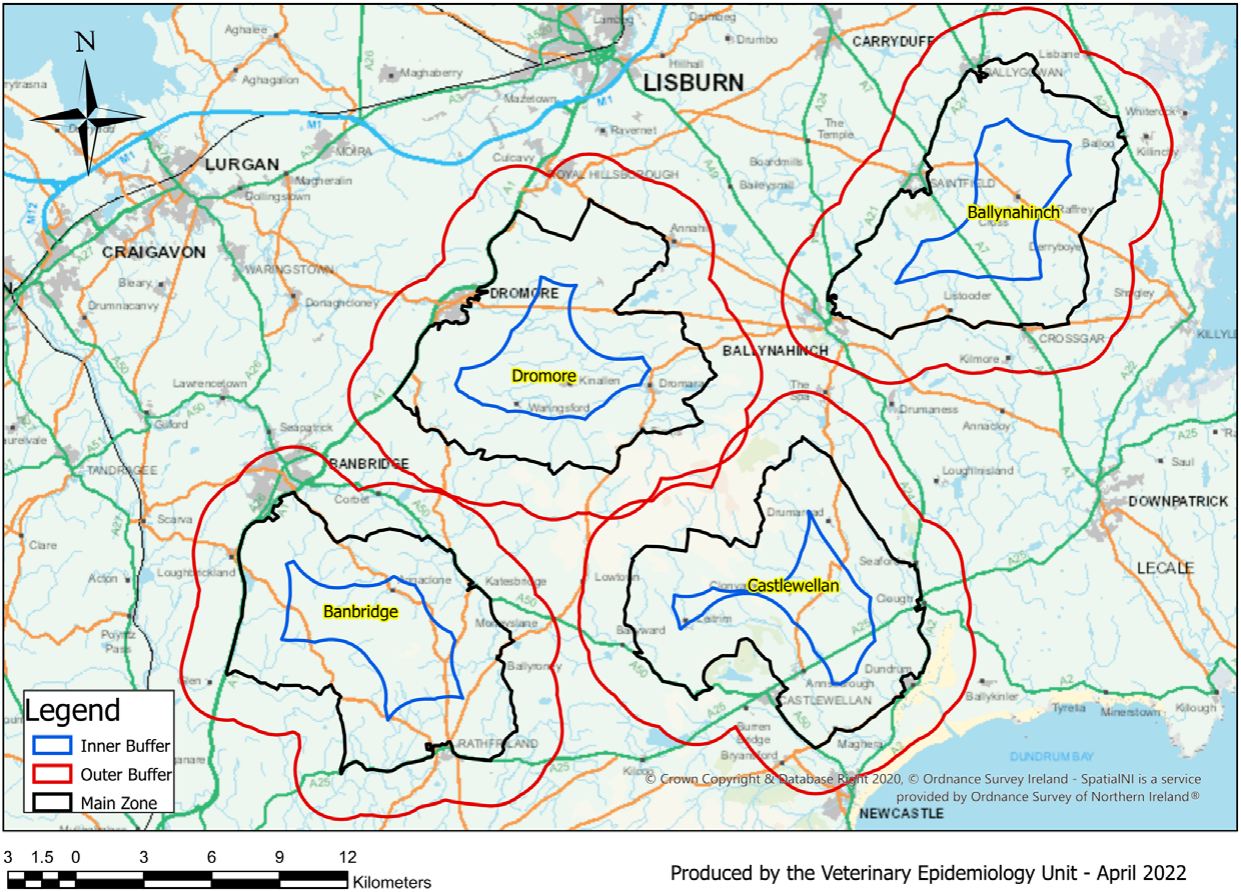
TVR area (Banbridge) and comparison areas (Ballynahinch, Castlewellan, Dromore) shown (black polygons) with each of their 2 km buffer areas (red polygons).

This observational study investigated incidence rates for cattle herd bTB breakdowns disclosed in the TVR treatment area (Banbridge) compared to three areas (Dromore, Ballynahinch, and Castlewellan) that did not receive any badger intervention (Figure 1). Also included in the study was a 2 km buffer zone around the outer edge of the four study areas. A multivariable Poisson regression was used to calculate the bTB herd incidence rate ratios (IRR) in the years 2011 to 2019, inclusive. Statistical analysis was carried out using R software [26]. Data used in this study was aggregated at the area and year level. This article has been written in compliance with the STROBE statement.

The three comparison areas were chosen using the same methodology (based on high cattle and active badger sett density in conjunction with having a relatively high confirmed bTB herd prevalence) as outlined for the TVR treatment area [23]. These areas were selected prior to the commencement of the TVR study, and they also had the advantage of being proximate to each other (Figure 1).

Each cattle herd in Northern Ireland has its location coordinates stored in a national database representative of its Centre of Activity (COA) along with details of its bTB testing history and cattle movements, including purchases [27]. The COA of a farm is where the main housing and land parcel exists; however, it takes no account of out farms (areas of land used by a farm business not directly attached to the main holding) or conacre (rented ground), which may be geographically distant from the COA. Farms with a COA located within areas used for this study were geolocated using ArcGIS software (ArcGIS [GIS software] Version 10.8.1, ESRI Inc., Redlands, CA 92373, USA). Each farm business in Northern Ireland claiming Basic Payment Scheme (BPS) completes a Field Data Sheet (FDS) that details a list of fields on which they carry out their activities. The FDS for each farm business in the TVR, comparison areas, and buffer zones allowed aggregation of field count and area data, providing a total of ground claimed both within the area and external to it. This aggregated FDS data for each business meant that each could have an overall percentage allocated to it representing the proportion of land it claimed in the given study area.

**Figure 2.**
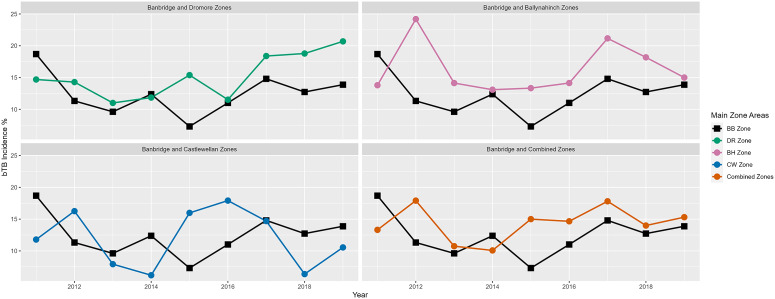
Comparison of bTB herd incidence (%) for new restrictions in Banbridge (BB) TVR area to Dromore (DR), Ballynahinch (BH), Castlewellan (CW), and the three areas combined in the years 2011 to 2019 inclusive.

**Figure 3.**
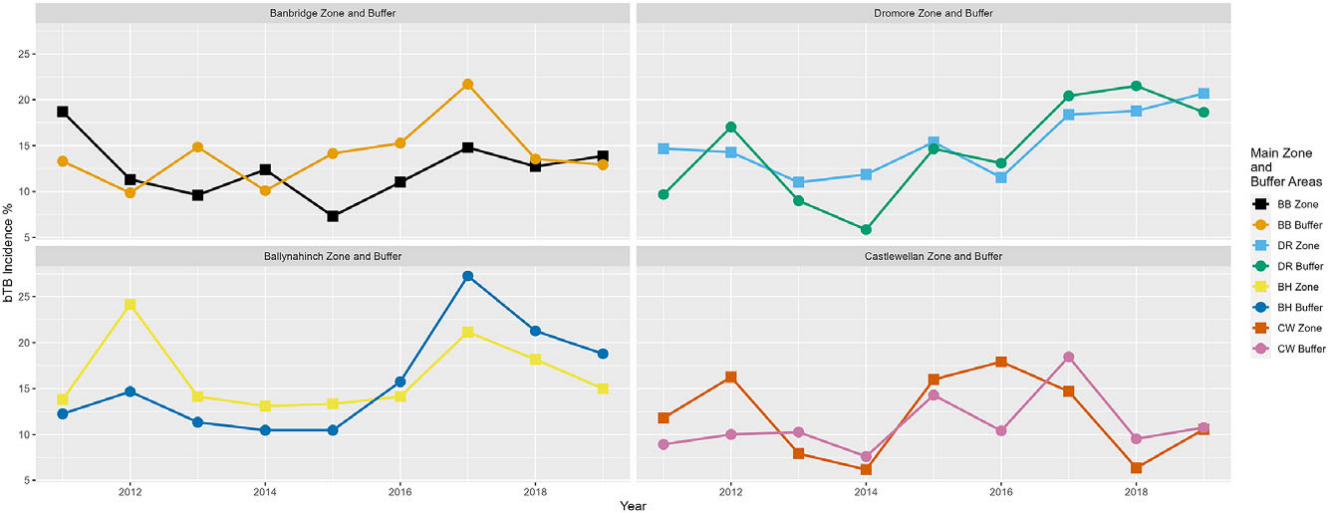
Comparison of bTB herd incidence (%) for new restrictions in each of the four areas Banbridge (BB) TVR area, Dromore (DR), Ballynahinch (BH), and Castlewellan (CW) to their buffer areas in the years 2011 to 2019 inclusive.

In terms of descriptive statistics, farm demographics within eight separate areas, the Banbridge TVR study area, the three comparison areas, and the four 2 km buffer zones during the years 2011 to 2019 have been presented in Table 1. Also, even though not the focus of this article (the focus was on the calculation of IRRs), Figures 2 and 3 have been added to include a graphical description of bTB herd incidence over time (2011 to 2019) in each of the relevant areas (the four study areas, the combined study areas, and the four study plus buffer areas).

**Table 1.** Median values (with interquartile range) for study farm demographics aggregated to area level in the years 2011 to 2019

Study area	Number of farm businesses	Herd size	Percentage dairy enterprises	Percentage of farms that purchased at least one bovine animal in previous year	Percentage farms with all their ground contained in the associated area
Banbridge TVR treatment area	214(210–220)	45(40–47)	22.4(22.0–22.9)	85.6(84.5–89.3)	53.4(53.1–55.6)
Dromore comparison area	211(210–217)	42.5(40–43)	20.5(20.3–20.9)	82.5(80.3–83.3)	52.1(51.7–52.4)
Ballynahinch comparison area	187(184–191)	40(39.5–41)	15.5(15.2–15.6)	79.9(78.3–80.1)	57.8(57.5–58.6)
Castlewellan comparison area	243(240–245)	33.5(32.5–35)	6.6(6.5–6.7)	78.8(78.0–79.9)	49.4(48.7–50.0)
Banbridge TVR 2 km buffer area	208(203–212)	43(41–44)	18.4(18.2–18.7)	83.3(81.1–84.7)	25.4(25.2–26.4)
Dromore 2 km buffer area	186(188–191)	42.5(40–43)	16.5(16.2–16.7)	82.8(78.0–83.8)	25.5(25.0–26.3)
Ballynahinch 2 km buffer area	147(143–150)	43(41–44)	14.3(14.0–14.7)	76.9(75.9–77.4)	24.1(22.0–26.0)
Castlewellan 2 km buffer area	160(158–168)	33.5(31–34)	9.3(8.9–9.5)	75.2(74.4–76.2)	22.5(22.4–23.6)

The response variable in this study was the IRR, determined by modelling the count of newly confirmed bTB herd breakdowns and using log time at risk as an offset explanatory variable. As the IRR values quoted in the results and discussion are in all cases adjusted for at least one or more of the study variables, they are adjusted IRRs. At a herd level, confirmation of bTB infection was deemed to have occurred when a Comparative Intradermal Tuberculin Test (CITT) positive animal was disclosed with visible bTB-like lesions at slaughter or there was disclosure of greater than one CITT-positive animal independent of lesion status. Confirmation was also deemed to have occurred when lymph nodes from an animal were confirmed as histological and/or bacteriological positive for bTB on laboratory examination. Time at risk was the total time in years when all cattle herds within a particular area were not restricted due to bTB. This was calculated by subtracting the aggregation of time periods during which confirmed bTB herd breakdowns had their Officially Tuberculosis Free (OTF) status removed from the total time in years for all herds within a particular area. Removal of OTF status meant a herd had its official capacity to sell animals to other herds withdrawn, its re-establishment only occurring when there was statutory compliance with bTB scheme rules.

There were 11 explanatory factors included; area (as described above); year (2011 to 2019 inclusive); number of farm businesses (defined as a cattle herd which carried out a CITT with one or more animals in the given year); total CITT herd tests (defined as a total of routine, risk, and restricted CITT herd tests); total number of CITT reactors and Lesions at Routine Slaughter (LRS) where bTB was confirmed, measured in a unit of per 50 animals; median herd size determined from annual bTB herd test data; percentage of herds which were defined as dairy enterprises (presence of a milk licence); percentage of farms which disclosed a confirmed bTB herd breakdown in the previous 2 years; percentage of farms which purchased any cattle in the previous year; and percentage of farms with a COA in a particular area which have all their associated ground claimed on BPS in that area. Also included as an explanatory variable for each area was the average number of active main badger setts per km^2^ (Table 2), which was extracted from data provided by Reid et al. [28]. These data are available in the supplementary material (Supplementary Table 1).

**Table 2.** Summary badger demographics for the TVR area (Banbridge) and comparison areas^a^

Study area	Average active main sett density (per km^2^)	Badger population point estimate based on literature estimate	Badger population point estimate based on genetic estimate
Banbridge TVR treatment area	1.11	535	634
Dromore comparison area	1.22	717	571
Ballynahinch comparison area	1.16	559	588
Castlewellan comparison area	1.05	500	597
Banbridge TVR 2 km buffer area	1.07	535	609
Dromore 2 km buffer area	1.17	689	556
Ballynahinch 2 km buffer area	1.16	589	634
Castlewellan 2 km buffer area	1.04	471	568

a Extracted from [28].

Analysis of the data in this study was carried out in two parts. The first part used Poisson regression (calculating an adjusted IRR for confirmed bTB breakdowns) comparing the TVR area (as baseline) individually to each of the three comparison areas along with an amalgamation of the three comparison areas (Table 3), and each of the four areas to their buffer zone in three separate model structures (Table 4). The three model structures used were area on its own, area included additively with year, and area as an interaction with year (2011 to 2019, inclusive). In each of these three model structures, the Likelihood Ratio Test (LRT) was used to select the best fit, determined by a statistical significance of *P* < 0.05, or in situations where more than one of the models had a *P* < 0.05, the model with the lowest *P*-value. In any case, where none of the three model structures were significant, the most parsimonious model (area only) was chosen.

**Table 3.** Dromore, Ballynahinch, and Castlewellan area adjusted IRRs (incidence rate ratios) compared to Banbridge TVR area (reference level) for years 2011 to 2019 inclusive

Area compared to Banbridge TVR area	Best fit model for IRR: Y ~ Area Y ~ Area + Year Y ~ Area × Year	IRR for Area	95% confidence interval for IRR		*P*-value
			Lower	Upper	
Dromore	Y ~ Area	1.30	1.10	1.53	<0.01
Ballynahinch	Y ~ Area	1.39	1.17	1.64	<0.01
Castlewellan	Y ~ Area	0.99	0.83	1.17	0.865
Dromore, Ballynahinch, Castlewellan combined	Y ~ Area	1.20	1.04	1.38	0.011

**Table 4.** Banbridge, Dromore, Ballynahinch, and Castlewellan area adjusted IRRs (incidence rate ratios), where comparison was made to their own individual buffer zones, used as the reference level for years 2011 to 2019 inclusive

Main area compared to buffer zone	Best fit model for IRR: Y ~ Area Y ~ Area + Year Y ~ Area × Year	IRR for Area	95% confidence interval for IRR		
			Lower	Upper	*P*-value
Banbridge	Y ~ Area	0.87	0.73	1.03	0.1
Dromore	Y ~ Area + Year	1.03	0.87	1.21	0.768
Ballynahinch	Y ~ Area + Year	1.04	0.88	1.25	0.627
Castlewellan	Y ~ Area	1.13	0.94	1.38	0.2

The second part of the analysis in this study also used Poisson regression, firstly applying univariate (Supplementary Tables 2 and 3) and then forward stepwise regression (Table 5) to all 11 explanatory variables (described above), starting with the Null model and adding variables until no further reduction in Akaike Information Criterion (AIC) could be achieved. The resulting model from the forward stepwise regression had its fit assessed using deviance and Pearson statistics [29]. Models were further tested for statistically significant overdispersion [29].

**Table 5.** Multivariable Poisson regression model selected from original 11 explanatory variables by forward stepwise regression for years 2011 to 2019 inclusive

Explanatory variable	IRR	Standard error	95% CI for IRR		*P*-value
			Lower	Upper	
Percentage of farms with a bTB breakdown in the previous 2 years	1.07	0.016	1.04	1.11	<0.01
Total number of CITT reactors and LRSs (per 50)	1.04	0.013	1.01	1.07	<0.01
Year	0.98	0.013	0.96	1.00	0.106

Note: See Supplementary Tables 2 and 3 for univariate analyses results for the 11 potential explanatory variables comparing the Banbridge TVR area with the Banbridge buffer area and comparing the Banbridge TVR area with the combined comparison areas Dromore, Ballynahinch, and Castlewellan in the years 2011 to 2019 inclusive.

## Results

Table 1 shows data on farm demographics within eight separate areas: the Banbridge TVR study area, the three comparison areas, and the four 2 km buffer zones during the years 2011 to 2019. In terms of number and size of herds, the main difference shown in Table 1 was between the Banbridge TVR study area and the Castlewellan comparison area, where Banbridge had a smaller number of larger herds (214 herds of median size 45 vs 243 herds of median size 33.5). In terms of buffer zones, the Banbridge buffer area was similar to the TVR study area in terms of the number and size of herds (214 herds of median size 45 vs 208 herds of median size 43). However, for Castlewellan, there were fewer herds in the buffer compared to its comparison area, probably because the buffer zone encroaches on an upland region (243 herds vs 160 herds). In terms of the percentage of dairy herds, the Banbridge TVR area was most similar to Dromore (22.4% vs 20.5%), with Castlewellan differing most at 6.6%. In all areas, the percentage of farms carrying out at least one bovine animal purchase per year was greater than 75%, with the highest level in the Banbridge TVR area (85.6%). In terms of the percentage of farms which had all their ground contained within an associated area, this ranges from 49.4% (Castlewellan) to 57.8% (Ballynahinch). Buffer areas, which were 2 km zones surrounding each area, had lower proportions of total ground associated with each farm business, ranging from 22.5% (Castlewellan buffer zone) to 25.5% (Dromore buffer zone).

Table 2 shows summary data on badger demographics within the areas that were derived from spatial data extracted from badger population estimates for Northern Ireland [27]. Average active main badger sett densities were similar across the eight areas (1.04–1.22/km^2^), with those assumed to equate to badger social group densities [23].

Figure 2 shows a line graph of bTB herd incidence percentage for new restrictions in the Banbridge TVR area compared to Dromore, Ballynahinch, Castlewellan, and the three areas combined in the years 2011 to 2019 inclusive. Figure 3 shows line graphs of bTB herd incidence percentage for new restrictions, but in this case, it compares each area to its buffer zone.

With the first part of the analysis, each of the three non-intervention areas (Dromore, Ballynahinch, Castlewellan, and the three areas combined) were compared in terms of adjusted IRR using Banbridge as the reference level (Table 3). The model structure chosen in each of these cases included only the area explanatory variable as this provided the optimum for analysis. The results show that at a 5% significance level, the comparison areas of Dromore, Ballynahinch, and the three areas combined all had statistically significant higher adjusted IRRs than the Banbridge TVR area: 1.30 (*P* < 0.01), 1.39 (*P* < 0.01), and 1.20 (*P* = 0.011), respectively. However, the Castlewellan area had an adjusted IRR which was not statistically significantly different from the Banbridge TVR area: 0.99 (*P* = 0.865).

In each of the four study areas (Banbridge, Dromore, Ballynahinch, and Castlewellan), the adjusted IRR was also calculated relative to their individual buffer zone (used as the reference level) in order to determine if rate differences between them were statistically significant at the 5% level (Table 4). The best fitting model structures in this case included Year as an additive explanatory factor for the Dromore and Ballynahinch areas. Results show that the adjusted IRRs comparing buffer zones to their own areas were not statistically significantly different in any of the four cases: Banbridge adjusted IRR = 0.87 (*P* = 0.1), Dromore adjusted IRR = 1.03 (*P* = 0.768), Ballynahinch adjusted IRR = 1.04 (*P* = 0.627), and Castlewellan adjusted IRR = 1.13 (*P* = 0.2).

In the second part of the analysis, the univariate results (Supplementary Tables 2 and 3) showed that the two variables with the strongest statistically significant association were the percentage of farms with a bTB herd breakdown in the previous 2 years and the total number of CITT reactors and LRSs (per 50 animals). Multivariable results from the Poisson forward stepwise regression resulted in a model containing three of the original variables: percentage of farms with a bTB herd breakdown in the previous 2 years, total number of CITT reactors, and LRSs (per 50 animals) and year (Table 5). The first variable added in the stepwise regression was the percentage of farms with a bTB herd breakdown in the previous 2 years with an AIC reduction of 62.08, followed by the total number of CITT reactors and LRSs (per 50 animals) with an AIC reduction of 6.05, and finally, the year with an AIC reduction of 0.6. The goodness of fit (gof) for this model was assessed using both deviance and Pearson residuals (*P* = 0.071 and *P* = 0.082, respectively). Dispersion for this model was calculated at a ratio value of 1.366 (*P* = 0.081), indicating that the level of dispersion was not statistically significant at the 5% level [25].

## Discussion

The ultimate purpose of any badger intervention such as TVR is the reduction of bTB incidence in the local cattle population, the premise being that lower bTB levels in badgers will lead to less onward transmission of infection to cattle. The TVR study provided field evidence of a statistically significant reduction in badger bTB prevalence, from 14% (95% CrI: 0.10–0.20) at the start to 1.9% (95% CrI: 0.8–3.8) after 5 years of TVR application [25]. Previous work in GB and ROI demonstrated that levels of bTB in cattle were lower in areas subjected to extensive proactive badger culling compared to matched control areas in which reactive culling or no culling was carried out (however, there was substantial variation in the results between replicates in these studies) [3–8]. Non-selective culling as applied in GB and ROI to reduce bTB incidence in a badger population differs from the TVR approach in that the field testing of badgers in TVR attempts to selectively remove infected (test positive) badgers and protect test-negative badgers by vaccination.

The TVR project took place in the Banbridge area (Figure 1) during the 5-year period from 2014 to 2018 inclusive. With the initial model constructed to carry out the analysis (Table 3), the explanatory variables of Area and Year were considered to determine if relative to the Banbridge TVR area, spatial or temporal effects were evident. The time period over which the adjusted IRR was calculated ranged from 2011 to 2019, capturing 3 years of data before the project started and 1 year after. This extended time range (2011 to 2019) provided the study with the mechanism to assess the temporal effect of the years 2014 to 2018 in which TVR was applied, in the Banbridge area. Extending the observation period after 2018 for longer than 1 year would have been preferable in this study in order to determine if there were any lagged bTB effects in cattle from the application of TVR. However, the disruption to bTB surveillance which occurred due to the COVID-19 pandemic in 2020 meant that comparisons to previous years would not have been valid. The approach is similar to that used previously by Brunton et al. [7] and Downs et al. [8].

With this study, the bTB herd adjusted IRR was greater (*P* < 0.05) in Dromore, Ballynahinch, and the combined areas, compared to Banbridge, but it did not differ from the adjusted IRR in Castlewellan. In terms of herd demographics, the area most analogous to Banbridge in terms of the number of herds, herd size, percentage dairy, purchasing practices, and percentage of land contained in the area was Dromore, with an adjusted IRR relative to Banbridge of 1.30 (95% CI: 1.10–1.53; *P* < 0.01). Castlewellan herd demographics were least similar to Banbridge for the three comparison areas used in this study as it contained a greater number of smaller herds and fewer dairy enterprises (Table 1).

In this study, potential explanatory variables were also applied in both univariate and multivariable models in order to determine which had the strongest association with the IRR (similar to the methodology employed by Downs et al. [8]). Results showed that the two variables with the strongest association with the IRR were a previous bTB herd breakdown within 2 years and the number of CITT reactors and LRSs (per 50 animals). Both the study area and the number of badger setts were used as explanatory variables when generating the multivariable model, but neither were included in the final model. The final multivariable model generated in this study points towards explanatory variables which could potentially encompass risk from multiple different sources, such as carryover of infection, contiguous spread, or purchase of infected animals as well as wildlife. In a separate genomic study based on *M. bovis* isolates from the Banbridge TVR area, it was found that transmission dynamics in this area appeared to be dominated by cattle-associated transmission (35/37 direct transmission events) with only sporadic inter-species transitions (2/37 direct transmission events) and no badger-to-badger direct transmission events being observed [12, 13]. While this may be true of the Banbridge locality, other studies have found contrasting transmission dynamics [15, 30], and it is suggested that there is regional heterogeneity in the epidemiology of bTB [12, 31, 32].

This study had one treatment area (Banbridge) of approximately 100 km^2^ and three local comparison areas (Dromore, Ballynahinch, and Castlewellan) of similar size where no treatment was applied. The median number of herds in the Banbridge treatment area during the study period was 214 (IQR: 210–220). Given these demographics, a simulation study was carried out to explore the power of routine bTB surveillance to assess the impact of the TVR intervention on bTB incidence in cattle herds [33]. This work showed that with a 60% reduction in bTB herd incidence over the 5-year period, the study would achieve an estimated power of 76% (below but approaching the minimum 80% power normally associated with scientific experiments). This meant the design of TVR only had the power to detect large reductions in incidence, and smaller reductions in incidence would be subject to low power values (in other words, a higher-powered study may have incorporated more variables into the model). As previously highlighted [23], the lack of treatment area replication was another limitation of this study.

Each of the areas (study and comparison) had a 2 km buffer zone allocated around its edge, and a relative bTB herd adjusted IRR was calculated for each buffer zone. One of the objectives of this study was to determine if TVR led to any perturbation effect [24]. In England, badger culling has been associated with an increased incidence of bTB in cattle herds surrounding the cull areas [34], something which has not been reported in ROI [3]. This effect in England is thought to be due to culling-induced perturbation of an otherwise stable badger population social structure, leading to increased ranging and thus more opportunity for *M. bovis* transmission [35]. A study carried out on badger home ranges in the Banbridge TVR area showed that neither annual nor monthly home ranges differed significantly in size between years, suggesting they were not significantly altered by the bTB intervention that was applied [24]. Furthermore, the stability of the badger social structure in the TVR area was verified using capture-recapture data along with a badger genetic relatedness study [36]. Results from this present study show no statistically significant difference in the bTB herd adjusted IRR of the 2 km buffer zone relative to the main TVR area, thus providing no evidence that a demonstrable perturbation effect exists when TVR was applied. However, given the above discussion on the power of the TVR study and the calculation that the design falls below the minimum of 80%, it could be argued that even if a statistically significant difference between the TVR area and its buffer area existed, it may not have been detected anyway.

While these findings (supported by other evidence [12, 13, 36]) indicated that badger-to-cattle transmission may be a rare event in this area, this does not necessarily imply that such events are inconsequential; given that such an introduction by badgers may lead to substantial multiplication of infection levels by onward cattle-to-cattle transmission [1]. Moreover, the introduction of a badger intervention into the TVR area may have empowered herd keepers to make behavioural changes that impacted on bTB transmission within the area, which are not linked to any effect of the actual intervention. Unfortunately, no attempt was made to assess herd keeper behavioural changes during the TVR study. Furthermore, any lag effect between the badger intervention and subsequent impact on bTB in cattle herds may have been censored through the time period considered in this analysis. Modelling evidence based on data from both NI and GB would suggest that any changes in cattle bTB herd breakdowns involving a badger intervention with a lethal component (proactive and selective culling) would have been observed although the timescales did extend to a 5-year period after intervention [37],

## Conclusion

This study used routine bTB cattle herd surveillance data to determine if the TVR intervention had any effect in reducing the infection at a herd level. Even though results showed that there was a statistically significant lower adjusted IRR in the Banbridge TVR area compared to two of the other three comparison areas, results from a multivariable model in this study and those obtained from other studies [12, 13, 35] would suggest that the main driver of bTB transmission in the Banbridge TVR area is cattle-to-cattle transmission. This predominance of cattle-to-cattle transmission in this intervention area may act to limit the effectiveness of any wildlife intervention applied in relation to bTB herd breakdowns. The other issue which must be considered when interpreting the results from this study is its scientific power, as this is below the recommended minimum of 80%, which means there are reduced chances of detecting statistically significant differences in the IRRs between the TVR and comparison areas. In our study, statistical significance was achieved in two cattle-related risk factors, but however, it cannot be ruled out that other potential risk factors may have also demonstrated significance in a larger study.

## Data Availability

The data that support the findings in this study are available in their aggregated form in Supplementary Table 1.

## Supplementary material

For supplementary material accompanying this paper visit http://doi.org/10.1017/S0950268823001061. click here to view supplementary material
