# Supplementary material for: Assessing the impact of a test and vaccinate or remove badger intervention project on bovine tuberculosis levels in cattle herds
Source: Epidemiol Infect. 2023 Jul 4;151:e115. doi: 10.1017/S0950268823001061 (PMC10369427; doi:10.1017/S0950268823001061)
Supplement: Supplementary file 1 [file hygsup.zip › S0950268823001061sup002.docx]

Supplementary Table 2. Univariate analysis for the 11 potential explanatory variables comparing the Banbridge TVR area with the Banbridge buffer area in the years 2011 to 2019 inclusive.

| **Explanatory Variable** | **IRR** | **Standard Error** | **95% CI for IRR** | | **P-Value** |
| --- | --- | --- | --- | --- | --- |
|  |  |  | **Lower** | **Upper** |  |
| **Area** | 0.87 | 0.088 | 0.73 | 1.03 | 0.100 |
| **Year** | 1.02 | 0.017 | 0.99 | 1.06 | 0.201 |
| **Number of farm businesses/herds** | 1.00 | 0.006 | 0.99 | 1.01 | 0.754 |
| **Total CITT herd tests** | 1.00 | 0.001 | 1.00 | 1.01 | 0.009 |
| **Total number of CITT reactors & confirmed LRS animals (per 50 animals)** | 1.15 | 0.032 | 1.08 | 1.21 | <0.001 |
| **Median herd size** | 1.03 | 0.014 | 1.00 | 1.06 | 0.028 |
| **% herds that were registered as dairy herds** | 0.96 | 0.022 | 0.92 | 1.00 | 0.054 |
| **% herds with a confirmed bTB herd breakdown in the previous 2 years** | 1.14 | 0.025 | 1.09 | 1.20 | <0.001 |
| **% herds that had purchased cattle during the previous years** | 0.98 | 0.012 | 0.96 | 1.00 | 0.075 |
| **% farms/herds with all their land within their associated 100km^2^ area** | 0.99 | 0.003 | 0.99 | 1.00 | 0.061 |
| **Average number of active main badger setts per km^2^** | 0.01 | 2.926 | 0.00 | 2.52 | 0.100 |

CITT = Comparative intradermal tuberculin test

LRS = lesion at routine slaughter (where bovine tuberculosis infection is confirmed).

Supplementary Table 3. Univariate analysis for the 11 potential explanatory variables comparing the Banbridge TVR area with the combined comparison areas Dromore, Ballynahinch and Castlewellan in the years 2011 to 2019 inclusive.

| **Explanatory Variable** | **IRR** | **Standard Error** | **95% CI for IRR** | | **P-Value** |
| --- | --- | --- | --- | --- | --- |
|  |  |  | **Lower** | **Upper** |  |
| **Area** | 1.20 | 0.072 | 1.04 | 1.38 | 0.011 |
| **Year** | 1.02 | 0.012 | 0.99 | 1.04 | 0.158 |
| **Number of farm businesses/herds** | 0.99 | 0.002 | 0.99 | 1.00 | <0.001 |
| **Total CITT herd tests** | 1.00 | 0.001 | 1.00 | 1.00 | <0.001 |
| **Total number of CITT reactors & confirmed LRS animals (per 50 animals)** | 1.08 | 0.010 | 1.06 | 1.10 | <0.001 |
| **Median herd size** | 1.02 | 0.006 | 1.01 | 1.03 | 0.002 |
| **% herds that were registered as dairy herds** | 1.01 | 0.005 | 1.00 | 1.02 | 0.157 |
| **% herds with a confirmed bTB herd breakdown in the previous 2 years** | 1.10 | 0.011 | 1.07 | 1.12 | <0.001 |
| **% herds that had purchased cattle during the previous years** | 0.99 | 0.007 | 0.98 | 1.01 | 0.243 |
| **% farms/herds with all their land within their associated 100km^2^ area** | 1.02 | 0.009 | 1.00 | 1.04 | 0.016 |
| **Average number of active main badger setts per km^2^** | 1.36 | 0.487 | 0.53 | 3.54 | 0.523 |

CITT = Comparative intradermal tuberculin test

LRS = lesion at routine slaughter (where bovine tuberculosis infection is confirmed).

Supplementary Figure 1: Percentage incidence of newly applied and removed confirmed restrictions along with percentage of confirmed restriction which span the entire calendar year for each of the years 2011 to 2019 inclusive. (BB-Banbridge, BH-Ballynahinch, CW-Castlewellan, DR-Dromore).


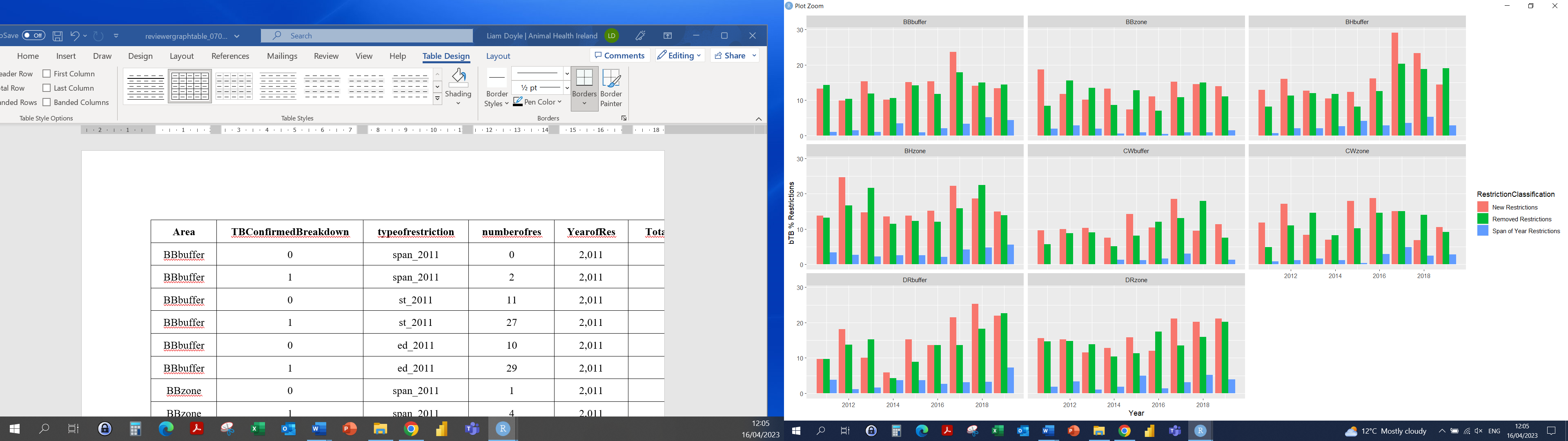


Supplementary Figure 2: Percentage incidence of newly applied and removed unconfirmed restrictions along with percentage of unconfirmed restriction which span the entire calendar year for each of the years 2011 to 2019 inclusive. (BB-Banbridge, BH-Ballynahinch, CW-Castlewellan, DR-Dromore).


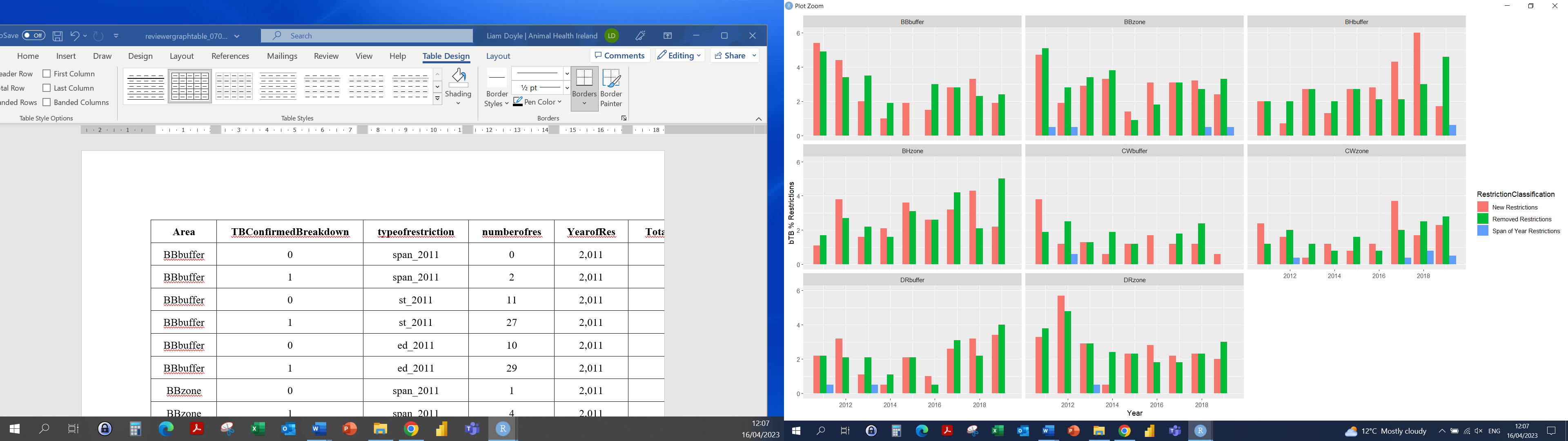


Supplementary Figure 1 and Supplementary Figure 2 show the percentage incidence of newly applied and removed restrictions along with percentage of restrictions which span the entire calendar year for each of the years 2011 to 2019 inclusive, in confirmed and unconfirmed bTB breakdowns. The bar charts show how percentages of restrictions applied, removed and those spanning the year change on yearly basis. The bars tend to show that in years with a high percentage of restrictions being applied there is also a high percentage of restriction removal. It is also a feature of Supplementary 1 with confirmed restrictions that consistent high incidence levels tend to raise the percentage of restrictions which span an entire year. With unconfirmed restrictions (Supplementary 2) there are fewer of these which span entire year periods reflecting the fact that these generally are shorter duration in nature.
