# Supplementary material for: Assessing the impact of a test and vaccinate or remove badger intervention project on bovine tuberculosis levels in cattle herds
Source: Epidemiol Infect. 2023 Jul 4;151:e115. doi: 10.1017/S0950268823001061 (PMC10369427; doi:10.1017/S0950268823001061)
Supplement: Supplementary file 1 [file hygsup.zip › S0950268823001061sup003.docx]

STROBE Statement for submitted manuscript:

Doyle, L.P., Gordon, A.W., Molloy, C., O’Hagan, M.J.H., Georgaki, A., Courcier, E.A., Harwood, R.G., Menzies F.D. (2023). Assessing impact of a Test and Vaccinate or Remove badger intervention project on levels of bovine tuberculosis. *Epidemiology & Infection*, (submitted).

|  | Item No. | Recommendation | Page  No. | Relevant text from manuscript |
| --- | --- | --- | --- | --- |
| **Title and abstract** | 1 | (*a*) Indicate the study’s design with a commonly used term in the title or the abstract | 2 | ‘This observational study’ |
|  |  | (*b*) Provide in the abstract an informative and balanced summary of what was done and what was found | 2 | ‘This observational study used routine bTB surveillance data of cattle to determine if the TVR intervention had any effect in reducing the infection at a herd level. The study design included the TVR treatment area (Banbridge) compared to the three adjacent 100 km^2^ areas (Dromore, Ballynahinch and Castlewellan) which did not receive any badger intervention. Results showed that there were statistically lower bTB herd incidence rate ratios in the Banbridge TVR area compared to two of the other three comparison areas but with bTB herd history and number of bTB infected cattle being the main explanatory variables along with Year. This finding is consistent with other study results conducted as part of the TVR project that suggested that the main transmission route for bTB in the area was cattle to cattle spread.’ |
| Introduction | | | |  |
| Background/rationale | 2 | Explain the scientific background and rationale for the investigation being reported | 3 | ‘‘Evidence of an association between *M. bovis* infection in cattle and badgers exists from a variety of sources.’ Summary findings from such studies are then presented.  ‘…two host bTB transmission system between badgers and cattle is a complex interaction probably varying between different localities, which is dependent on a range of badger and cattle metrics unique to a particular area, but with higher transmission rates within species than between species.’  ‘The Test and Vaccinate or Remove (TVR) project was a five year badger intervention (2014-2018) in a 100km^2^ area of County Down, Northern Ireland.’ Summary findings on badgers from this project are then presented. |
| Objectives | 3 | State specific objectives, including any prespecified hypotheses | 4 | ‘The primary objective of this study was to assess if the application of a TVR intervention in the 100km^2^ core area had an impact on the level of bTB in the local cattle herds compared with similar sized non-treatment areas in the same locality over the same time period. It was also an objective of this study to determine if application of TVR intervention led to any increase in bTB among herds adjacent to the treatment area (perturbation effect). This part of the objective was met by comparing the bTB herd incidence in a two km buffer zone around the TVR intervention with similar buffer zones around the comparison areas.’ |
| Methods | | | |  |
| Study design | 4 | Present key elements of study design early in the paper | 4 | ‘This observational study investigated incidence rates for cattle herd bTB breakdowns disclosed in the TVR treatment area (Banbridge) compared to three areas (Dromore, Ballynahinch and Castlewellan) that did not receive any badger intervention (Figure 1). Also included in the study was a two km buffer zone around the outer edge of the four study areas. Multivariable Poisson regression was used to calculate the bTB herd incidence rate ratios (IRR) in the years 2011 to 2019, inclusive. Statistical analysis was carried out using R software [26]. Data used in this study were aggregated at the area and year level. …..  The response variable in this study was the IRR, determined by modelling the count of newly confirmed bTB herd breakdowns and using log time at risk as an offset explanatory variable.’ |
| Setting | 5 | Describe the setting, locations, and relevant dates, including periods of recruitment, exposure, follow-up, and data collection | 2-4 | See above extracts. Also, Figure 1. |
| Participants | 6 | (*a*) *Cohort study*—Give the eligibility criteria, and the sources and methods of selection of participants. Describe methods of follow-up  *Case-control study*—Give the eligibility criteria, and the sources and methods of case ascertainment and control selection. Give the rationale for the choice of cases and controls  *Cross-sectional study*—Give the eligibility criteria, and the sources and methods of selection of participants | 4 | ‘This observational study investigated incidence rates for cattle herd bTB breakdowns disclosed in the TVR treatment area (Banbridge) compared to three areas (Dromore, Ballynahinch and Castlewellan) that did not receive any badger intervention (Figure 1). Also included in the study was a two km buffer zone around the outer edge of the four study areas. ….  Each cattle herd in Northern Ireland has its location coordinates stored in a national database [27] representative of its Centre of Activity (COA). The COA of a farm is where the main housing and land parcel exists ..’ |
|  |  | (*b*) *Cohort study*—For matched studies, give matching criteria and number of exposed and unexposed  *Case-control study*—For matched studies, give matching criteria and the number of controls per case | Not applicable |  |
| Variables | 7 | Clearly define all outcomes, exposures, predictors, potential confounders, and effect modifiers. Give diagnostic criteria, if applicable | 4-5 | ‘The response variable in this study was the IRR, determined by modelling the count of newly confirmed bTB herd breakdowns and using log time at risk as an offset explanatory variable. As the IRR values quoted in the results and discussion are in all cases adjusted for at least one or more of the study variables they are adjusted IRRs. At a herd level, confirmation of bTB infection was deemed to have occurred when a Comparative Intradermal Tuberculin Test (CITT) positive animal was disclosed with visible bTB like lesions at slaughter or there was disclosure of greater than one CITT positive animal independent of lesion status. Confirmation was also deemed to have occurred when lymph nodes from an animal were confirmed as histological and/or bacteriological positive for bTB on laboratory examination. Time at risk was the total time in years all herds within a particular area were not restricted due to bTB. This was calculated by subtracting the aggregation of time periods during which confirmed bTB herd breakdowns had their Officially Tuberculosis Free (OTF) status removed from the total time in years for all herds within a particular area. Removal of OTF status meant a herd had its official capacity to sell animals to other herds withdrawn, its re-establishment only occurring when there was statutory compliance with bTB scheme rules. …..  There were eleven explanatory factors included; area (as described above), year (2011 to 2019 inclusive), number of farm businesses (defined as a cattle herd which carried out a CITT with one or more animals in the given year), total CITT herd tests (defined as a total of routine, risk and restricted CITT herd tests), total number of CITT reactors and Lesions at Routine Slaughter (LRS) where bTB was confirmed, measured in a unit of per 50 animals, median herd size determined from annual bTB herd test data, percentage of herds which were defined as dairy enterprises (presence of a milk licence), percentage of farms which disclosed a confirmed bTB herd breakdown in the previous two years, percentage of farms which purchased any cattle in the previous year and percentage of farms with a COA in a particular area which have all their associated ground claimed on BPS in that area. Also included as an explanatory variable for each area was the average number of active main badger setts per km^2^ (Table 2) , which was extracted from data provided by Reid et al. [28].’ |
| Data sources/ measurement | 8* | For each variable of interest, give sources of data and details of methods of assessment (measurement). Describe comparability of assessment methods if there is more than one group | 4-5 | ‘The three comparison areas were chosen using the same methodology (based on high cattle and active badger sett density in conjunction with having a relatively high confirmed bTB herd prevalence) as outlined for TVR treatment area [23]. These areas were selected prior to the commencement of the TVR study and they also had the advantage of being proximate to each other (Figure 1).’  ‘Each cattle herd in Northern Ireland has its location coordinates stored in a national database along with details of its bTB testing history and cattle movements including purchases [27].’  ‘ …. the average number of active main badger setts per km^2^ (Table 2) , which was extracted from data provided by Reid et al. [28].’ |
| Bias | 9 | Describe any efforts to address potential sources of bias | 4 | ‘The three comparison areas were chosen using the same methodology (based on high cattle and active badger sett density in conjunction with having a relatively high confirmed bTB herd prevalence) as outlined for TVR treatment area [23]. These areas were selected prior to the commencement of the TVR study and they also had the advantage of being proximate to each other (Figure 1).’ |
| Study size | 10 | Explain how the study size was arrived at | 3-4, 8 | ‘The Test and Vaccinate or Remove (TVR) project was a five year badger intervention (2014-2018) in a 100km^2^ area of County Down, Northern Ireland [23]’ …  ‘This observational study investigated incidence rates for cattle herd bTB breakdowns disclosed in the TVR treatment area (Banbridge) compared to three areas (Dromore, Ballynahinch and Castlewellan) that did not receive any badger intervention (Figure 1).’ ….  ‘This study had one treatment area (Banbridge) of approximately 100km^2^ and three local comparison areas (Dromore, Ballynahinch and Castlewellan) of similar size where no treatment was applied. The median number of herds in the Banbridge treatment area during the study period was 214 (IQR: 210-220). Given these demographics, a simulation study was carried out to explore the power of routine bTB surveillance to assess the impact of the TVR intervention on bTB incidence in cattle herds [33]. This work showed that with a 60% reduction in bTB herd incidence over the five year period the study would achieve an estimated power of 76% (below but approaching the minimum 80% power normally associated with scientific experiments).’ |

Continued on next page

| Quantitative variables | 11 | Explain how quantitative variables were handled in the analyses. If applicable, describe which groupings were chosen and why | 4-5 | See 7 b& 8 above. |
| --- | --- | --- | --- | --- |
| Statistical methods | 12 | (*a*) Describe all statistical methods, including those used to control for confounding | 5 | ‘Analysis of the data in this study was carried out in two parts. The first part used Poisson regression (calculating an adjusted IRR for confirmed bTB breakdowns) comparing the TVR area (as baseline) individually to each of the three comparison areas along with an amalgamation of the three comparison areas (Table 3), and each of the four areas to their buffer zone in three separate model structures (Table 4). The three model structures used were area on its own, area included additively with year and area as an interaction with year (2011 to 2019, inclusive). In each of these three model structures, the Likelihood Ratio Test (LRT) was used to select the best fit, determined by a statistical significance of P<0.05, or in situations where more than one of the models had a P<0.05, the model with the lowest P-value. In any case, where none of the three model structures were significant the most parsimonious model (area only) was chosen.  The second part of the analysis in this study also used Poisson regression, firstly applying univariate (Supplementary Tables 2 and 3) and then forward stepwise regression (Table 5) to all eleven explanatory variables (described above); starting with the Null model and adding variables until no further reduction in Akaike Information Criterion (AIC) could be achieved. The resulting model from the forward stepwise regression had its fit assessed using deviance and Pearson statistics [29]. Models were further tested for statistically significant overdispersion [29]. ‘ |
|  |  | (*b*) Describe any methods used to examine subgroups and interactions | Not applicable |  |
|  |  | (*c*) Explain how missing data were addressed | Not applicable |  |
|  |  | (*d*) *Cohort study*—If applicable, explain how loss to follow-up was addressed  *Case-control study*—If applicable, explain how matching of cases and controls was addressed  *Cross-sectional study*—If applicable, describe analytical methods taking account of sampling strategy | Not applicable |  |
|  |  | (*e*) Describe any sensitivity analyses | Not applicable |  |
| Results | | | | |
| Participants | 13* | (a) Report numbers of individuals at each stage of study—eg numbers potentially eligible, examined for eligibility, confirmed eligible, included in the study, completing follow-up, and analysed | 6. Tables 1 & 2. Supplementary Table 1 | ‘Table 1 shows data on farm demographics within eight separate areas, the Banbridge TVR study area, the three comparison areas and the four 2km buffer zones during the years 2011 to 2019. In terms of number and size of herds the main difference shown in Table 1 was the between the Banbridge TVR study area and the Castlewellan comparison area where Banbridge had a smaller number of larger herds (214 herds of median size 45 vs 243 herds of median size 33.5). In terms of buffer zones, the Banbridge buffer area was similar to the TVR study area in terms of number and size of herds (214 herds of median size 45 vs 208 herds of median size 43). However, for Castlewellan there were less herds in the buffer compared to its comparison area, probably because the buffer zone encroaches on an upland region (243 herds vs 160 herds). In terms of percentage of dairy herds, the Banbridge TVR area was most similar to Dromore (22.4% vs 20.5%) with Castlewellan differing by 6.6%. In all areas the percentage of farms carrying out at least one bovine animal purchase per year was greater than 75% with the highest level in the Banbridge TVR area (85.6%). In terms of the percentage of farms which had all their ground contained within an associated area, this ranges from 49.4% (Castlewellan) to 57.8% (Ballynahinch). Buffer areas, which were two km zones surrounding each area, had lower proportions of total ground associated to each farm business ranging from 22.5% (Castlewellan buffer zone) to 25.5% (Dromore buffer zone).  Table 2 shows summary data on badger demographics within the areas that was derived from spatial data extracted from badger population estimates for Northern Ireland [27]. Average active main badger sett densities were similar across the eight areas (1.04-1.22 / km^2^) with those assumed to equate to badger social group densities [23]. |
|  |  | (b) Give reasons for non-participation at each stage | Not applicable / no cattle in herd |  |
|  |  | (c) Consider use of a flow diagram | Not applicable |  |
| Descriptive data | 14* | (a) Give characteristics of study participants (eg demographic, clinical, social) and information on exposures and potential confounders | 6. Tables 1 & 2. Supplementary Table 1 | See 13(a) above |
|  |  | (b) Indicate number of participants with missing data for each variable of interest | Not applicable |  |
|  |  | (c) *Cohort study*—Summarise follow-up time (eg, average and total amount) | Not applicable |  |
| Outcome data | 15* | *Cohort study*—Report numbers of outcome events or summary measures over time |  |  |
|  |  | *Case-control study—*Report numbers in each exposure category, or summary measures of exposure |  |  |
|  |  | *Cross-sectional study—*Report numbers of outcome events or summary measures | 6-7.  Tables 3-5  Supplementary Tables 2 & 3 |  |
| Main results | 16 | (*a*) Give unadjusted estimates and, if applicable, confounder-adjusted estimates and their precision (eg, 95% confidence interval). Make clear which confounders were adjusted for and why they were included | 6-7.  Tables 3-5 | ‘With the first part of the analysis , each of the three non-intervention areas (Dromore, Ballynahinch, Castlewellan and the three areas combined) were compared in terms of adjusted IRR using Banbridge as the reference level (Table 3). The model structure chosen in each of these cases included only the area explanatory variable as this provided the optimum for analysis. The results show that at a 5% significance level, the comparison areas of Dromore, Ballynahinch and the three areas combined all had statistically significant higher adjusted IRRs than the Banbridge TVR area; 1.30 (P<0.01), 1.39 (P<0.01) and 1.20 (P=0.011), respectively. However, the Castlewellan area had an adjusted IRR which was not statistically significantly different from the Banbridge TVR area; 0.99 (P=0.865).  In each of the four study areas (Banbridge, Dromore, Ballynahinch and Castlewellan), the adjusted IRR was calculated relative to their individual buffer zone (used as the reference level) in order to determine if rate differences between them were statistically significant at the 5% level (Table 4). Best fitting model structures in this case included year as an additive explanatory factor for Dromore and Ballynahinch areas. Results show that the adjusted IRRs comparing buffer zones to their own areas were not statistically significantly different in any of the four cases; Banbridge adjusted IRR=0.87 (P=0.1), Dromore adjusted IRR=1.03 (P=0.768), Ballynahinch adjusted IRR=1.04 (P=0.627) and Castlewellan adjusted IRR=1.13 (P=0.2).  In the second part of the analysis the univariate results (Supplementary Tables 2 and 3) showed that the two variables with the strongest statistically significant association were percentage of farms with a bTB herd breakdown in the previous two years and total number of CITT reactors and LRSs (per 50 animals). Multivariable results from Poisson forward stepwise regression resulted in a model containing three of the original variables; percentage of farms with a bTB herd breakdown in the previous two years, total number of CITT reactors and LRSs (per 50 animals) and year (Table 5). The first variable added in the stepwise regression was percentage of farms with a bTB herd breakdown in the previous two years with an AIC reduction of 62.08, followed by total number of CITT reactors and LRSs (per 50 animals) with an AIC reduction of 6.05 and finally, year with an AIC reduction of 0.6. The goodness of fit (gof) for this model was assessed using both deviance and Pearson residuals (P=0.071 and P=0.082, respectively). Dispersion for this model was calculated at a ratio value of 1.366 (P=0.081) indicating that the level of dispersion was not statistically significant at the 5% level [[2](#_ENREF_24)5].’ |
|  |  | (*b*) Report category boundaries when continuous variables were categorized | Not applicable |  |
|  |  | (*c*) If relevant, consider translating estimates of relative risk into absolute risk for a meaningful time period | Not applicable |  |

Continued on next page

| Other analyses | 17 | Report other analyses done—eg analyses of subgroups and interactions, and sensitivity analyses | Not applicable |  |
| --- | --- | --- | --- | --- |
| Discussion | | | | |
| Key results | 18 | Summarise key results with reference to study objectives | 8 & 9 | ‘In this study, potential explanatory variables were also applied in both univariate and multivariable models in order to determine which had strongest association to the IRR (similar to the methodology employed by Downs et al. [8]). Results showed that the two variables with strongest association to the IRR were a previous bTB herd breakdown within two years and number of CITT reactors and LRSs (per 50 animals). Both the study area and number of badger setts were used as explanatory variables when generating the multivariable model but neither were included in the final model. The final multivariable model generated in this study points towards explanatory variables which could potentially encompass risk from multiple different sources, such as carryover of infection, contiguous spread or purchase of infected animals as well as wildlife’ …  ‘Results from this present study show no statistically significant difference in the bTB herd adjusted IRR of the 2km buffer zone relative to the main TVR area, thus providing no evidence that a demonstrable perturbation effect exists when TVR was applied.’ |
| …Limitations | 19 | Discuss limitations of the study, taking into account sources of potential bias or imprecision. Discuss both direction and magnitude of any potential bias | 8-9 | ‘Given these demographics, a simulation study was carried out to explore the power of routine bTB surveillance to assess the impact of the TVR intervention on bTB incidence in cattle herds [33]. This work showed that with a 60% reduction in bTB herd incidence over the five year period the study would achieve an estimated power of 76% (below but approaching the minimum 80% power normally associated with scientific experiments). This meant the design of TVR only had the power to detect large reductions in incidence and smaller reductions in incidence would be subject to low power values (in other words, a higher powered study may have incorporated more variables into the model). As previously highlighted [23], lack of treatment area replication was another limitation of this study.’ ….  ‘.. introduction of a badger intervention into the TVR area may have empowered herd keepers to make behavioural changes that impacted on bTB transmission within the area, which are not linked to any effect of the actual intervention. Unfortunately, no attempt was made to assess herd keeper behavioural changes during the TVR study. Furthermore, any lag effect between the badger intervention and subsequent impact on bTB in cattle herds may have been censored through the time period considered in this analysis.’ |
| Interpretation | 20 | Give a cautious overall interpretation of results considering objectives, limitations, multiplicity of analyses, results from similar studies, and other relevant evidence | 8-9 | ‘The final multivariable model generated in this study points towards explanatory variables which could potentially encompass risk from multiple different sources, such as carryover of infection, contiguous spread or purchase of infected animals as well as wildlife. In a separate genomic study based on *M. bovis* isolates from the Banbridge TVR area, it was found that transmission dynamics in this area appeared to be dominated by cattle associated transmission (35/37 direct transmission events) with only sporadic inter-species transitions (2/37 direct transmission events) and no badger-to-badger direct transmission events being observed [[12](#_ENREF_12),13]. While this may be true of the Banbridge locality, other studies have found contrasting transmission dynamics [15, 30] and it is suggested that there is regional heterogeneity in the epidemiology of bTB [12, 31-32].’ …  ‘One of the objectives of this study was to determine if TVR led to any perturbation effect [24]. In England, badger culling has been associated with an increased incidence of bTB in cattle herds surrounding the cull areas [34] something which has not been reported in ROI [[3](#_ENREF_3)]. This effect in England is thought to be due to culling induced perturbation of an otherwise stable badger population social structure, leading to increased ranging and thus more opportunity for *M. bovis* transmission [35]. A study carried out on badger home ranges in the Banbridge TVR area showed that neither annual nor monthly home ranges differed significantly in size between years, suggesting they were not significantly altered by the bTB intervention that was applied [24]. Furthermore, stability of the badger social structure in the TVR was verified using capture-recapture data along with badger genetic relatedness study [36]. Results from this present study show no statistically significant difference in the bTB herd adjusted IRR of the 2km buffer zone relative to the main TVR area, thus providing no evidence that a demonstrable perturbation effect exists when TVR was applied.’ …  ‘While these findings (supported by other evidence [12, 13, 36]) indicated that badger to cattle transmission may be a rare event in this area, this does not necessarily imply that such events are inconsequential; given that such an introduction by badgers may lead to substantial multiplication of infection levels by onward cattle to cattle transmission [1]. ‘ |
| Generalisability | 21 | Discuss the generalisability (external validity) of the study results | 8 | ‘..While this may be true of the Banbridge locality, other studies have found contrasting transmission dynamics [15, 30] and it is suggested that there is regional heterogeneity in the epidemiology of bTB [12, 31-32].’ |
| Other information | |  | | |
| Funding | 22 | Give the source of funding and the role of the funders for the present study and, if applicable, for the original study on which the present article is based | 10 | ‘Financial support  This research received no specific grant from any funding agency, commercial or not-for-profit sectors.’ |

*Give information separately for cases and controls in case-control studies and, if applicable, for exposed and unexposed groups in cohort and cross-sectional studies.

**Note:** An Explanation and Elaboration article discusses each checklist item and gives methodological background and published examples of transparent reporting. The STROBE checklist is best used in conjunction with this article (freely available on the Web sites of PLoS Medicine at http://www.plosmedicine.org/, Annals of Internal Medicine at http://www.annals.org/, and Epidemiology at http://www.epidem.com/). Information on the STROBE Initiative is available at www.strobe-statement.org.
